# L-Alliin Modulates Brain Region-Specific Neuroinflammatory Responses to Lipopolysaccharide in Diet-Induced Obese Mice

**DOI:** 10.3390/brainsci16020243

**Published:** 2026-02-22

**Authors:** Celia González-Castillo, Daniel Ortuño-Sahagún, Carolina Guzmán-Brambila, Daniel Ulises Torres-Reyes, Lucrecia Carrera-Quintanar, Oscar Arias-Carrión

**Affiliations:** 1 Tecnologico de Monterrey, Escuela de Medicina, Campus Guadalajara, Zapopan 45201, Mexico; celia.glz@tec.mx (C.G.-C.); caro@tec.mx (C.G.-B.); 2 Laboratorio de Neuroinmunobiología Molecular, Instituto de Neurociencias Traslacionales, CUCS, Universidad de Guadalajara, Guadalajara 44350, Mexico; daniel.ortuno@academicos.udg.mx; 3 Doctorado en Ciencias de la Nutrición Traslacional, Departamento de Alimentación y Nutrición, Centro Universitario de Ciencias de la Salud, Universidad de Guadalajara, Guadalajara 44340, Mexico; daniel.torres@academicos.udg.mx; 4 Instituto de Investigación en Cáncer en la Infancia y Adolescencia, Departamento de Clínicas de la Reproducción Humana, Crecimiento y Desarrollo Infantil, Centro Universitario de Ciencias de la Salud, Universidad de Guadalajara, Guadalajara 44340, Mexico; 5 División de Neurociencias Clínica, Instituto Nacional de Rehabilitación Luis Guillermo Ibarra Ibarra, Mexico City 14389, Mexico; 6 Tecnologico de Monterrey, Escuela de Medicina y Ciencias de la Salud, Mexico City 14380, Mexico

**Keywords:** neuroinflammation, high-fat diet, cytokines, hypothalamus, hippocampus, organosulfur compounds

## Abstract

**Highlights:**

**What are the main findings?**

- A high-fat diet markedly amplifies LPS-induced neuroinflammatory responses in a brain-region-specific manner, especially in the frontal cortex and hypothalamus.
- L-alliin significantly reduces cytokine overactivation, with stronger anti-inflammatory effects in metabolically stressed (HFD-fed) animals.

**What are the implications of the main findings?**

- L-alliin acts as a context-dependent modulator, selectively normalizing cytokine pathways according to the metabolic load and neural region involved.
- These results support the therapeutic potential of L-alliin for targeting obesity-related neuroinflammation and highlight the need for region-specific strategies in CNS immunometabolic disorders.

**Abstract:**

Background/Objectives: A high-fat diet disrupts metabolic and neuroimmune balance in the brain, making neural tissue more reactive to inflammatory challenges. However, it is not well understood how this vulnerability varies across brain regions or how natural anti-inflammatory compounds influence it. Methods: In this study, we examined how the garlic-derived molecule L-alliin modulates the inflammatory response triggered by lipopolysaccharide in the frontal cortex, hippocampus, and hypothalamus of mice fed either a standard or high-fat diet. Results: Measurements of cytokine gene expression showed that the high-fat diet greatly increased the inflammatory response in the frontal cortex and hypothalamus, with the hypothalamus displaying the strongest overall activation. Treatment with L-alliin significantly reduced elevated cytokine levels in both regions, with the reductions most pronounced in animals on the high-fat diet. In contrast, the hippocampus showed a distinct pattern: expression of TNF-α and IL-1β changed very little across diets or treatments, whereas IL-6 and CCL2 were selectively altered by L-alliin, depending on the animals’ metabolic state. Conclusions: These findings demonstrate that diet-induced obesity does not affect the entire brain uniformly. Instead, inflammatory pathways are altered region-specifically, and L-alliin modulates these pathways with sensitivity to both brain region and metabolic condition. This work emphasizes the importance of accounting for neuroanatomical differences when developing strategies to reduce inflammation in obesity-associated conditions.

## 1. Introduction

High-fat diet (HFD) consumption is now recognized as a potent disruptor of central nervous system (CNS) homeostasis, initiating metabolic, vascular, and neuroimmune alterations that collectively sensitize the brain to inflammatory stimuli [1,2,3,4]. Sustained HFD exposure induces profound cognitive dysfunction, reduces synaptic integrity, and compromises blood–brain barrier (BBB) function, with effects particularly notable in hippocampal and cortical regions [2,5,6]. Even acute HFD exposure is sufficient to increase BBB permeability within one hour, with region-specific vascular leakage underscoring the brain’s rapid metabolic vulnerability [2]. Collectively, these studies identify a HFD as a systemic driver of neurometabolic stress, lowering the threshold for CNS inflammatory activation.

A central mechanistic component of this vulnerability is neuroinflammation [1,4,7,8,9]. A HFD elevates circulating lipopolysaccharide (LPS) and primes immune cells through chronic low-grade inflammation, amplifying cytokine responses to subsequent immune challenges [8,10]. Both experimental and clinical evidence show exaggerated induction of IL-1β, IL-6, TNF-α, and chemokines in the cortex and hippocampus after HFD exposure [5,6]. These cytokines are central regulators of leukocyte recruitment, neuronal excitability, glial activation, and BBB integrity; their dysregulation accelerates cognitive decline and metabolic–immune deterioration [11]. Furthermore, HFD-induced BBB disruption increases brain access to circulating inflammatory mediators, compounding regional susceptibility to cytokine overactivation.

Within this context, natural immunomodulators have attracted interest as potential modulators of neuroinflammation [5,10,12,13]. Alliin, the principal organosulfur compound in garlic, has been documented to possess anti-inflammatory and immunoregulatory properties. Recent evidence demonstrates that L-alliin modulates systemic cytokine production in diet-induced obesity (DIO), attenuates IL-1β–dependent cascades, and influences chemotactic responses such as MCP-1/CCL2 [10]. These effects occur alongside broader metabolic and neuroprotective actions described in preclinical obesity models, including modulation of hypothalamic inflammation, microglial remodeling, and neuronal vulnerability [3]. Nevertheless, despite growing evidence for L-alliin’s systemic immunoregulatory capacity, its region-specific effects within the brain—particularly under combined chronic metabolic stress and acute inflammatory challenge—remain undefined.

Brain regions differ in metabolic load, glial density, vascular specialization, and inflammatory thresholds [14,15]. The hypothalamus serves as a metabolic–immune interface and exhibits accelerated inflammatory responses under a HFD [16], while the hippocampus is more susceptible to structural and cognitive consequences [17]. The frontal cortex, by contrast, integrates metabolic, cognitive, and vascular signals and demonstrates robust IL-6 modulation in HFD models [5]. Given these well-established regional differences, determining whether L-alliin uniformly attenuates neuroinflammation or instead modulates discrete cytokine programs across brain regions is critical for understanding its therapeutic potential. By evaluating the frontal cortex, hippocampus, and hypothalamus in parallel, this study moves beyond the traditional model of uniform neuroimmune suppression. Unlike previous single-region or systemic assessments, our multi-regional approach allows us to analyze whether L-alliin exerts a broad inhibitory effect or instead functions as a specific modulator, recalibrating discrete cytokine programs based on the unique inducibility thresholds and metabolic sensitivities of specific brain circuits.

Here, we investigated how L-alliin shapes the acute neuroinflammatory response to LPS in mice fed a standard diet (STD) or a HFD, focusing on IL-1β, IL-6, TNF-α, and CCL2 expression in the frontal cortex, hippocampus, and hypothalamus. This approach allows us to determine whether L-alliin exerts broad anti-inflammatory activity, regionally selective modulation, or metabolic-state-dependent effects. Our findings reveal distinct regional architectures of cytokine responsiveness and position L-alliin as a non-uniform, context-dependent modulator of CNS inflammation.

## 2. Materials and Methods

### 2.1. Animals, Diets and Experimental Design

Male C57BL/6J mice (5 weeks old; *n* = 60) were housed under controlled temperature and humidity on a 12 h light/dark cycle with ad libitum access to food and water. The standard diet (STD) contains the following: 4.07 kcal/g, containing 18.3% protein, 22.1% fat, and 59.6% carbohydrate, from TestDiet™ (Richmond, VA, USA), diet 5755. The high-fat diet (HFD) contains the following: 5.1 kcal/g, containing 18.1% protein, 61.6% fat, and 20.3% carbohydrate, from TestDiet™ (Richmond, VA, USA), diet 58Y1.

After a 4-week acclimation period on a STD, animals were randomly assigned to four groups using Decision Analyst STATS 2.0^TM^ (Arlington, TX, USA) (*n* = 15 per group): STD + vehicle, STD + L-alliin, HFD + vehicle, and HFD + L-alliin. HFD feeding (12 weeks; TestDiet™ 58Y1; 61.6% kcal from fat) induced obesity, while control animals remained on a STD (TestDiet™ 5755; 22.1% kcal from fat). Inclusion criteria: male C57BL/6J mice; body weight 20 to 25 g; 7–8 weeks of age; health certificate. Exclusion criteria: other mouse strains; female C57BL/6J mice; body weight below 20 g or above 25 g; presence of infection. All procedures followed institutional guidelines and were approved by the Ethics Committee for Research and Biosafety of the Centro Universitario de Ciencias de la Salud, Universidad de Guadalajara (approval CI-02521 CUCS UdG).

### 2.2. L-Alliin Administration

L-alliin (≥90% purity, Sigma-Aldrich, St. Louis, MO, USA, catalog number 7426) was freshly dissolved in sterile 0.9% saline and administered by oral gavage (20 mg·kg^−1^) once daily for 4 consecutive weeks. Vehicle groups received matched volumes of saline. The selection of 20 mg·kg^−1^ as the optimal oral dose was based on extensive prior research by our group [10,18,19], including a series of in vitro and in vivo dose–response studies evaluating L-alliin at concentrations of 1, 5, 10, and 15 mg·kg^−1^. Although the lower doses demonstrated partial immunomodulatory activity, our findings indicated that the 20 mg·kg^−1^ dose produced the most robust and consistent systemic anti-inflammatory effect and neuroprotection in the context of diet-induced obesity (DIO). This dose was previously validated for its capacity to attenuate IL-1β-dependent cascades and modulate hypothalamic inflammation, establishing it as the most effective concentration for the current regional CNS analysis.

### 2.3. Acute Inflammatory Challenge and Tissue Collection

To evoke a standardized innate immune response, all animals received an intraperitoneal injection of LPS (*E. coli* 0111:B4; 5 mg·kg^−1^) (Millipore Sigma, Burlington, MA, USA) 60 min prior to sacrifice. The intraperitoneal administration was deliberately chosen to elicit a robust, standardized acute innate immune response in our HFD obesity model, enabling clear detection of region-specific cytokine modulation by L-alliin in metabolically stressed CNS circuits. We used the LPS challenge model to elicit an acute response and assess the tissue’s maximum inflammatory capacity, using the STD group as the induction baseline. Importantly, our experimental design involves sacrificing exactly 60 min post-LPS, capturing the early phase of innate immune activation while preventing progression to sublethal septic shock or prolonged systemic compromise. This short timeframe distinguishes our approach from depression-like or chronic neuroinflammation models, where lower doses (typically 0.5–1 mg/kg) are preferred to allow behavioral assessment at 24 h or beyond. Mice were euthanized by rapid decapitation to minimize confounding transcriptional changes associated with anesthetic exposure. Brains were quickly removed, placed on an ice-cooled platform, and microdissected to isolate the frontal cortex, hippocampus, and hypothalamus. Tissues were snap-frozen in liquid nitrogen and stored at −80 °C.

### 2.4. RNA Isolation and Quantitative PCR

Total RNA was extracted using silica-membrane spin-column purification. RNA concentration and purity were assessed by spectrophotometry. cDNA was synthesized with a reverse transcription kit according to the manufacturer’s instructions. Quantitative PCR (qPCR) was performed using SYBR Green chemistry on a real-time thermocycler. Primer pairs were validated for efficiency and amplicon specificity. Expression levels of IL-1β, IL-6, TNF-α, and CCL2 were quantified in each brain region. β-actin (ACTB) served as the reference gene for cortex and hypothalamus; ACTB or GAPDH was used in the hippocampus, depending on primer set compatibility. Relative expression values were calculated using the 2^−ΔΔCt method.

### 2.5. Statistical Analysis

Each brain region was analyzed independently. Group differences were assessed by one-way ANOVA followed by post hoc multiple-comparisons testing. In addition, a two-way ANOVA analysis was performed (Supplementary File S1). The analysis was performed using summary statistics (mean ΔCT ± SD) for each group (*n* = 10 per group) and using β-actin as the reference. Significance thresholds correspond to the notation used in figures: * *p* < 0.05; ** *p* < 0.01; *** *p* < 0.001; **** *p* < 0.0001. No animals or data points were excluded. Investigators were blinded to group assignment during qPCR quantification and analysis.

## 3. Results

### 3.1. Mice Weight and Food Consumption

At baseline (week 0), body weight was similar across all experimental groups (STD + V: 21.8 ± 1.3 g; STD + A: 21.5 ± 1.0 g; DRG + V: 22.3 ± 1.4 g; DRG + A: 22.5 ± 1.3 g; *p* > 0.05). After 12 weeks of dietary intervention, mice fed the high-fat diet showed a marked increase in body weight compared with standard-diet mice (DRG + V: 48.8 ± 5.8 g; DRG + A: 45.6 ± 4.9 g vs. STD + V: 27.9 ± 1.7 g; STD + A: 27.7 ± 1.6 g; *p* < 0.001), confirming successful induction of diet-induced obesity, while standard-diet mice exhibited only physiological weight gain. After four weeks of L-alliin administration (20 mg·kg^−1^), no significant differences in body weight were observed between L-alliin–treated and vehicle-treated groups within the same dietary condition (week 16: STD + V: 29.2 ± 2.0 g vs. STD + A: 29.1 ± 1.5 g; DRG + V: 51.2 ± 6.2 g vs. DRG + A: 48.0 ± 4.2 g; *p* > 0.05), indicating that body weight differences were due to diet composition rather than L-alliin treatment.

During weeks 0–12, food intake was similar across groups (STD + V: 21.4 ± 1.6 g; STD + A: 24.0 ± 1.3 g; DRG + V: 20.3 ± 4.1 g; DRG + A: 20.3 ± 4.1 g; *p* > 0.05). After L-alliin administration (weeks 12–16), intake decreased in all groups. Standard-diet mice receiving vehicle showed a non-significant reduction (21.4 ± 1.6 g to 19.2 ± 0.4 g; *p* > 0.05), while L-alliin–treated standard-diet mice exhibited a significant decrease (24.0 ± 1.3 g to 18.3 ± 2.9 g; *p* < 0.05). High-fat diet–fed mice also reduced intake over time (DRG + V: 20.3 ± 4.1 g to 17.1 ± 1.2 g; DRG + A: 20.3 ± 4.1 g to 16.2 ± 1.0 g), with no significant differences between vehicle and L-alliin treatment (*p* > 0.05), indicating that L-alliin significantly affected food intake only under standard dietary conditions.

### 3.2. The Frontal Cortex Exhibits a Globally Amplified Inflammatory Program Under a HFD That L-Alliin Uniformly Suppresses

**Figure 1:**
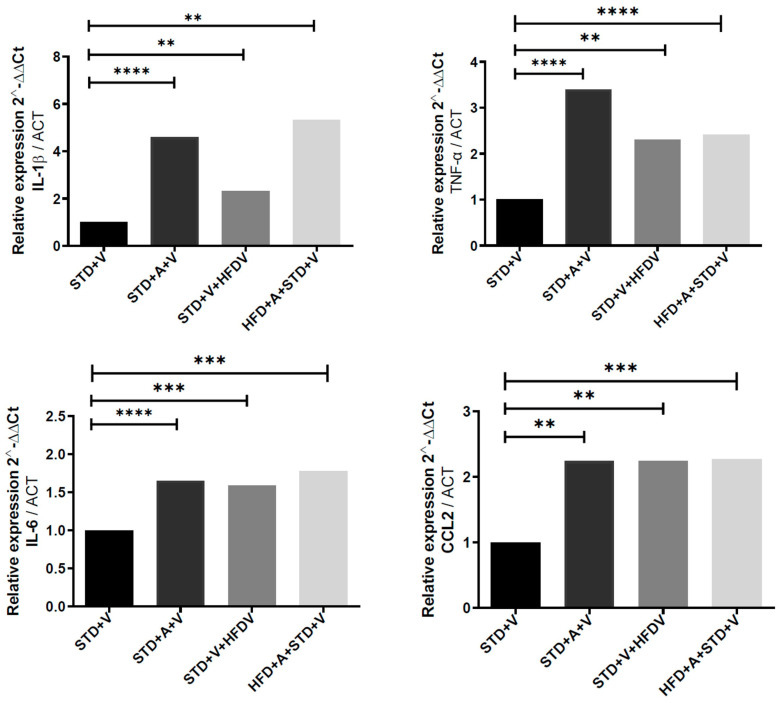
L-alliin modulates LPS-induced cortical cytokine expression in a diet-dependent manner. Relative mRNA expression of IL-1β, TNF-α, IL-6, and CCL2 in the frontal cortex of mice maintained on a standard diet (STD) or high-fat diet (HFD) and treated with vehicle (V) or L-alliin (A). All animals received an acute LPS challenge (5 mg·kg^−1^, i.p.) 60 min before tissue collection. Data are presented as relative expression (2^−^ΔΔCt) normalized to ACTB. The HFD markedly amplified LPS-evoked cytokine induction across all markers, whereas L-alliin significantly reduced expression (with specific increases per gene: IL-1β, 4.34-fold; IL-6, 0.78-fold; TNF-α, 1.4-fold; and CCL2, 1.27-fold), with the strongest suppressive effects observed in HFD-fed animals. One-way ANOVA determined statistical significance with post hoc multiple-comparisons testing. ** *p* < 0.01, *** *p* < 0.001, **** *p* < 0.0001.

The transcriptional architecture of the frontal cortex displayed three defining properties that distinguish its response to LPS and metabolic state (Figure 1). First, all four quantified cytokines—IL-1β, IL-6, TNF-α, and CCL2—were elevated by the combination of a HFD and LPS (with specific increases per gene: IL-1β, 4.34-fold; IL-6, 0.78-fold; TNF-α, 1.4-fold; and CCL2, 1.27-fold), producing the most robust neuroinflammatory burst among the three brain regions examined. This amplification was consistently greater than that observed under the STD. Second, L-alliin imposed a coordinated and broad suppression of this transcriptional program. In contrast to the hippocampus and hypothalamus, where L-alliin exerted more selective effects, the cortex exhibited uniform downregulation across all markers, with reductions spanning IL-1β (), IL-6 (), TNF-α (**), and CCL2 (*). Third, the magnitude of cytokine suppression scaled with metabolic load: L-alliin produced its strongest effects in HFD-fed mice, reversing a substantial portion of the HFD-driven upregulation. Together, these features indicate that the frontal cortex is both the most metabolically sensitized region and the region where L-alliin exerts the most globally coherent anti-inflammatory effect.

### 3.3. The Hippocampus Exhibits Selective Cytokine Responsiveness, Maintaining TNF-α Stability While Reallocating Inflammatory Weight Toward IL-6 and CCL2

**Figure 2:**
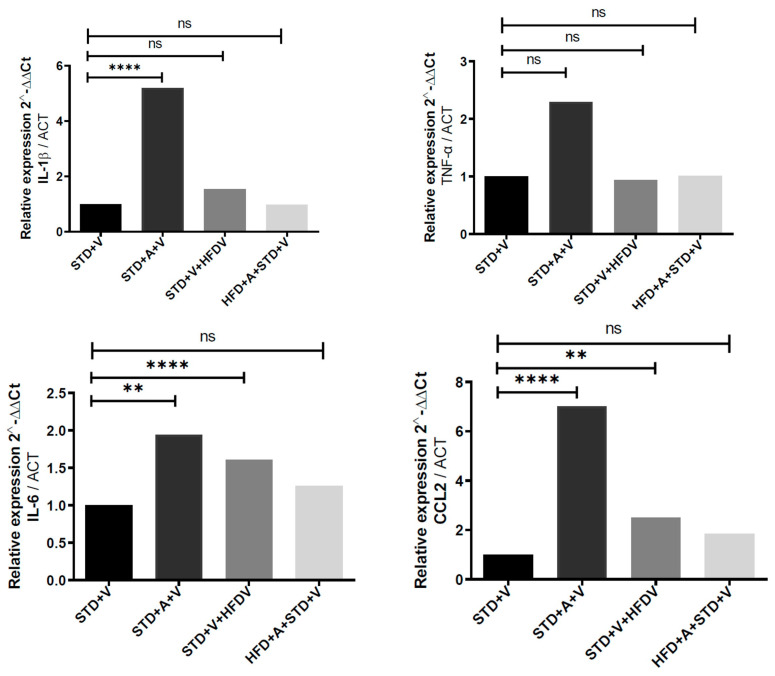
L-alliin selectively modulates hippocampal cytokine expression following acute LPS challenge in standard- and high-fat–fed mice. Relative mRNA expression of IL-1β, TNF-α, IL-6, and CCL2 in the hippocampus of mice maintained on a standard diet (STD) or high-fat diet (HFD) and treated with vehicle (V) or L-alliin (A). All animals received LPS (5 mg·kg^−1^, i.p.) 60 min before tissue collection. Expression values (2^−^ΔΔCt) were normalized to ACTB. Unlike the cortex and hypothalamus, hippocampal TNF-α and IL-1β expression remained largely unchanged across dietary and treatment conditions, indicating a constrained inflammatory response in this region. In contrast, IL-6 and CCL2 displayed significant responsiveness to L-alliin, with STD-fed mice showing robust cytokine elevation after LPS that L-alliin attenuated, whereas HFD-fed animals exhibited reduced or absent L-alliin effects. One-way ANOVA with post hoc multiple-comparisons testing was used to determine statistical significance. ** *p* < 0.01, **** *p* < 0.0001; ns = not significant.

The hippocampus differed from the cortex in three critical respects (Figure 2). First, TNF-α expression remained stable across all conditions—STD vs. HFD, vehicle vs. L-alliin—indicating that hippocampal TNF-α operates under a homeostatic constraint resistant to both diet-induced priming and pharmacologic modulation. This contrasts sharply with the cortex and hypothalamus, where TNF-α is highly labile. Second, IL-6 emerged as the principal modifiable cytokine. LPS induced IL-6 in both diets, and L-alliin significantly attenuated expression in STD (*p*) and more robustly in HFD-fed mice (**** *p*), revealing preferential targeting of IL-6 regulatory pathways in this region. Third, CCL2 displayed a distinctive bidirectional profile: elevated by L-alliin in STD mice (****), with a 3.38-fold increase in gene expression; however, this was unchanged under a HFD. This pattern suggests that metabolic state determines whether CCL2 is suppressible or permissive to L-alliin’s action. Thus, unlike the cortex, which undergoes global inflammatory suppression, the hippocampus reorganizes selectively, maintaining TNF-α invariance while permitting L-alliin to redirect IL-6 and CCL2 signaling.

### 3.4. The Hypothalamus Shows the Highest Metabolic Amplification and the Deepest L-Alliin-Mediated Repression Across Cytokines

**Figure 3:**
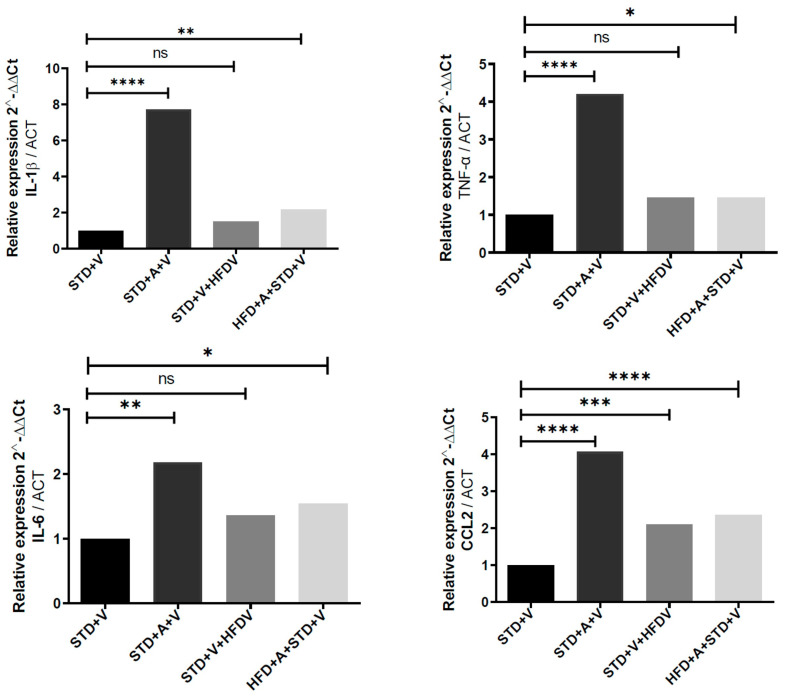
L-alliin robustly attenuates LPS-induced hypothalamic cytokine induction in a diet-dependent manner. Relative mRNA expression of IL-1β, TNF-α, IL-6, and CCL2 in the hypothalamus of mice fed a standard diet (STD) or high-fat diet (HFD) and treated with vehicle (V) or L-alliin (A). All animals received LPS (5 mg·kg^−1^, i.p.) 60 min before sacrifice. Cytokine expression (2^−^ΔΔCt) was normalized to ACTB. The hypothalamus showed the greatest overall cytokine induction after LPS, particularly for IL-1β and TNF-α, consistent with its heightened metabolic–immune sensitivity. L-alliin significantly reduced the expression of all measured cytokines, with the greatest suppression in HFD-fed mice, indicating enhanced responsiveness to metabolic stress. One-way ANOVA determined statistical significance with post hoc multiple-comparisons. * *p* < 0.05, ** *p* < 0.01, *** *p* < 0.001, **** *p* < 0.0001; ns = not significant.

The hypothalamus displayed three distinct structural features in its cytokine response (Figure 3). First, the HFD caused the greatest amplification of LPS-evoked transcriptional responses for IL-1β, IL-6, and TNF-α compared to the cortex and hippocampus, consistent with the hypothalamus’s role as a metabolic-immune integrator. IL-1β and TNF-α reached their highest absolute expression levels in this region. Second, L-alliin induced strong repression of inducible cytokines, particularly IL-1β and TNF-α (****), indicating that this region is the most pharmacologically responsive to L-alliin. Third, IL-6 suppression followed a graded, diet-dependent pattern: substantial in STD () and partial in HFD (*–) mice. This suggests that chronic metabolic stress does not eliminate sensitivity to L-alliin but instead alters its amplitude. Together, these features reveal that the hypothalamus is the most metabolically destabilized region under a HFD and the region where L-alliin restores cytokine balance most effectively.

### 3.5. Two-Way ANOVA of the Three Brain Regions Comparing the Effects of Diet and Treatment

The inflammatory response, as measured by gene expression, varies across brain regions. The hippocampus shows the greatest interactions between diet and treatment, indicating that obesity (HFD) modifies the effect of L-alliin in this region more than in other regions. For example, for IL-1β and IL-6, the interaction suggests that L-alliin may reduce expression in STD mice but have opposite or negligible effects in HFD mice in the hippocampus (Table 1).

Treatment with L-alliin consistently lowers gene expression (a higher ΔCT indicates lower expression; since we analyzed ΔCT, a lower mean ΔCT indicates suppression). This effect is significant in the hypothalamus for IL-1β and in the cortex for TNF-α, with borderline effects in other cases. These findings suggest that L-alliin has anti-inflammatory potential, particularly in obese mice for certain genes, but the effect is modest and region-dependent. There is no strong main effect of diet alone across regions, indicating that obesity does not uniformly elevate these inflammatory markers after LPS challenge in all brain areas. There is a borderline effect in the hippocampus for IL-1β (*p* = 0.051), where a HFD may alter baseline expression.

## 4. Discussion

The present work reveals that neuroinflammation in the metabolically stressed brain is not uniform but is instead regionally partitioned and circuit-specific. By mapping how L-alliin reshapes cytokine expression in the frontal cortex, hippocampus, and hypothalamus, our findings challenge the common assumption that nutraceutical anti-inflammatory agents operate through uniform suppression. Instead, L-alliin may function as a specific modulator, engaging inflammatory networks in ways that reflect each region’s metabolic load, cellular composition, and susceptibility to diet-induced immune priming.

### 4.1. A HFD Establishes a Primed Inflammatory Landscape That Amplifies LPS Responsivity

Across multiple models, exposure to a HFD recalibrates brain physiology at metabolic, vascular, and immune levels. Behavioral and biochemical studies demonstrate that a HFD impairs learning, disrupts hippocampal and cortical homeostasis, and elevates pro-inflammatory cytokines such as IL-6 [1,5,17]. Complementary evidence indicates that a HFD compromises BBB integrity, allowing abnormal molecular flux even after *one hour* of exposure [2]. These observations converge with findings that a HFD exacerbates cognitive deficits, BBB disruption, and cytokine disturbances through metabolic and gut–brain–immune mechanisms [6].

Our results are consistent with the broader literature: a HFD significantly increased cortical and hypothalamic IL-1β, IL-6, TNF-α, and CCL2 after LPS, revealing a global amplification of innate immune signaling. Chronic diet-induced inflammation thus appears to sensitize brain circuits to acute immune challenges, positioning the HFD as an upstream driver of exaggerated neuroinflammatory responses.

This priming effect is mediated by a marked shift in microglial phenotype toward a stressed or senescent M1-like state, leaving these resident immune cells chronically poised for hyper-reactivity upon a “second hit” such as LPS exposure [20,21]. Specifically, the global elevation of TNF-α is functionally significant; while its constitutive glial presence maintains essential homeostatic synaptic strength, HFD-induced upregulation places circuits—particularly the hippocampus—at increased risk for maladaptive TNFR1-mediated synaptic disruption [21,22]. This sensitization reflects a profound recalibration of the CNS, transforming a transient protective response into an exaggerated, potentially neurotoxic inflammatory storm.

### 4.2. L-Alliin Suppresses Neuroinflammation Where Metabolic Load Is Highest

In the cortex and hypothalamus—the regions most destabilized by the HFD—L-alliin exerted the strongest suppression of IL-1β and TNF-α. These effects parallel systemic findings that L-alliin attenuates IL-1β–dependent cascades and reshapes chemotactic signaling in diet-induced obesity [10]. Together, these results suggest that L-alliin disrupts inflammatory amplification loops that arise under metabolic stress.

The hypothalamus, in particular, showed both the strongest inflammatory activation under the HFD and the most pronounced normalization by L-alliin. This supports the view that hypothalamic circuits function as metabolic–immune integrators and are especially susceptible to obesity-induced neuroinflammation, microglial remodeling, and neuronal injury [10].

This increased vulnerability results from a critical molecular cascade: chronic exposure to HFD-derived free fatty acids and inflammatory mediators activates the IKK-β/NF-κB pathway in hypothalamic neurons and glial cells. This key mechanism promotes energy imbalance and obesity-associated sympathetic hyperactivity [10,20]. Additionally, HFD-induced systemic inflammation primes the central innate immune system by stimulating microglial activation through the Toll-like Receptor 4 (TLR4) pathway [23]. Thus, the global amplification of cytokine signaling (IL-1β, TNF-α) observed in the cortex and hypothalamus after LPS represents an acute exacerbation of this pre-existing, TLR4-sensitized, NF-κB-activated state [24]. L-alliin’s potent anti-inflammatory action is therefore likely achieved by blocking this central metabolic-inflammatory cascade, effectively neutralizing the key molecular architect of exacerbated neuroinflammatory responsivity [10].

### 4.3. Hippocampal TNF-α Invariance Reveals a Constrained Inflammatory Architecture

In contrast to the cortex and hypothalamus, hippocampal TNF-α remained strikingly stable across diets and treatments. This invariance is unexpected given the region’s sensitivity to diet-induced cognitive and structural impairments [5,6]. The finding suggests that TNF-α transcription in the hippocampus may be governed by distinct regulatory constraints, possibly related to cell-type composition, microglial maturation state, or local synaptic demands [25].

The finding that hippocampal TNF-α expression remains stable across all conditions is striking, particularly given the lability of this cytokine in the cortex and hypothalamus. While this suggests a regionally partitioned response characterized by distinct inducibility thresholds [10], alternative interpretations must be addressed. The 60 min sacrifice window may not have coincided with the peak of hippocampal TNF-α transcription, suggesting a different temporal kinetic compared to the metabolic–immune interface of the hypothalamus, especially given that acute dietary stressors can induce regional vascular leakage within this timeframe [2]. Furthermore, the use of bulk tissue qPCR may have introduced a cell-type dilution effect, masking subtle microglial changes. Nevertheless, this invariance aligns with the hypothesis that the hippocampus maintains tight homeostatic control over TNF-α to preserve synaptic integrity and plasticity, as this cytokine is a critical regulator of synaptic strength via microglia-dependent mechanisms [25]. Although these findings provide a detailed map of the regional transcriptional response, a limitation of this study is that the analysis focused exclusively on mRNA levels measured by qPCR. Further research is needed to determine whether these changes are reflected proportionately in protein levels and in specific functional or behavioural outcomes.

The specific cellular composition of the hippocampal niche appears to confer significant resilience: studies on chronic systemic inflammation have shown that microglial populations in the dentate gyrus (DG) maintain robust homeostatic gene expression and actively resist phenotypic activation [26], thereby constraining the transcriptional trigger for TNF-α overproduction. This inherent microglial constraint likely protects hippocampal circuitry from the systemic inflammatory priming observed in other regions [26]. Furthermore, this tight regulation is necessary because hippocampal function requires precise control; the cytokine mediates highly constrained, pathway-selective metaplasticity in the CA1 region, a process that depends on finely tuned local signaling rather than global amplification, which would lead to widespread synaptotoxicity [25,27]

Rather than broad suppression, L-alliin modulated selective pathways in the hippocampus: IL-6 and CCL2. These cytokines are closely linked to microglial activation, synaptic plasticity, and neurovascular signaling [28]. The bidirectional effects of L-alliin on CCL2—strong attenuation in the STD but not the HFD—highlight a deeper functional principle: the metabolic state determines the direction of L-alliin’s action.

This region-specific pattern aligns with emerging evidence that cytokine networks function as topologically distinct modules across brain regions, each with unique inducibility thresholds and metabolic sensitivities [10].

### 4.4. New Model of Neuroimmune Regulation

The findings presented here represent a conceptual departure from prior studies that characterized L-alliin solely as a general anti-inflammatory agent in systemic or hypothalamic contexts [3,10,29]. Our results demonstrate that metabolic history dictates inflammatory amplitude in a region-specific manner. A defining novelty is the hippocampal TNF-α invariance, suggesting that some brain regions resist systemic priming due to inherent cellular constraints [22,25]. This resilience, possibly mediated by specific microglial populations in the dentate gyrus that maintain homeostatic gene expression despite chronic systemic stress [26], reveals that L-alliin does not merely suppress inflammation but recalibrates cytokine networks in line with the local vascular and cellular architecture.

Interactions are strongest in the hippocampus (e.g., for IL-1β, IL-6, CCL2), indicating synergistic or antagonistic effects between obesity and L-alliin. In the cortex, TNF-α interaction suggests that L-alliin is more effective in one diet group. No clear interactions are observed in the hypothalamus, implying additive effects.

Overall, L-alliin appears to attenuate LPS-induced inflammation (lower IL-1β and TNF-α in some regions), but its efficacy depends on obesity status and brain region. The hippocampus may be more sensitive to the combined effects of obesity and treatment, which could have cognitive implications in obesity. These findings support the utility of a 2 × 2 design in revealing interactions that may be missed by one-way ANOVA. There is no evidence of broad systemic effects; region-specific targeting may be important for therapeutic applications.

Taken together, our results, along with prior literature, support an updated, regionally resolved framework for understanding diet-induced neuroinflammation [30]. Specifically, the data suggest that: (a) Metabolic history dictates inflammatory amplitude. Chronic HFD exposure establishes a pre-inflamed neural milieu that magnifies cytokine reactivity in both the cortex and hypothalamus, consistent with studies showing diet-driven increases in neuroinflammatory tone and cognitive vulnerability [5,6]. (b) BBB dysfunction enables exaggerated cytokine responses. Even short-term HFD exposure increases BBB permeability and allows greater access of circulating inflammatory signals to the brain, lowering the threshold for cytokine induction [2]. (c) L-alliin modulates inflammatory circuits according to their metabolic burden. Its strongest suppressive effects occur in regions where the HFD most disrupts neuroimmune signaling—paralleling systemic findings that L-alliin dampens IL-1β-driven inflammatory cascades in obese models [10]. (d) Cytokine networks are anatomically segregated. The hippocampus maintains TNF-α invariance while modulating diet-dependent IL-6 and CCL2, underscoring that neuroinflammation is not a monolithic phenomenon but a set of region-specific transcriptional programs with distinct inducibility thresholds.

Within this framework, L-alliin functions not as a generalized anti-inflammatory compound but as a circuit-tuned regulator, recalibrating cytokine programs in ways that reflect the metabolic, vascular, and cellular architecture of each brain region. This perspective positions L-alliin as a promising targeted intervention for metabolic neuroinflammation, where therapeutic success depends on respecting the intrinsic heterogeneity of CNS immune responsiveness.

Although direct assessments of BBB integrity were not performed, the clinical potential of L-alliin is supported by its interaction with the neurovascular unit. High-fat diets are known to trigger rapid BBB disruption, increasing the molecular flux of circulating inflammatory signals into the brain. The robust central effects observed here suggest that L-alliin can effectively reach or influence the CNS during these periods of vulnerability, potentially acting as a substrate for the LAT1 transporter at the blood–brain barrier. Thus, L-alliin may mitigate the inflammatory cascades that typically follow metabolically driven vascular leakage.

From a translational perspective, the clinical potential of L-alliin is supported by its favorable pharmacokinetic profile and specific mechanisms for brain entry. Pharmacokinetic studies in rodent models indicate that L-alliin is rapidly absorbed via the intestinal amino acid transport pathway [20,23], reaching peak blood concentrations (Tmax) within 10 min, with approximately 85.5% excreted within 72 h [10]. While it is primarily absorbed unchanged, it can also be partially metabolized into bioactive compounds such as allyl sulfenic acid and diallyl disulfide [26].

Crucially, L-alliin has been identified as a substrate for the Large Neutral Amino Acid Transporter 1 (LAT1/SLC7A5), a key transporter at the blood–brain barrier [22,25]. This suggests a targeted mechanism for CNS entry even under physiological conditions. However, our findings are particularly relevant in the context of metabolic stress, where HFD-induced BBB permeability increases within 1 h of exposure [17,30], potentially facilitating the influx of L-alliin or its metabolites into the brain at the time of neuroinflammatory priming [2,16].

Finally, translational feasibility is bolstered by recent clinical evidence demonstrating the safety and accumulation of L-alliin in human tissues, positioning this garlic-derived nutraceutical as a viable, low-toxicity candidate for long-term immunometabolic intervention [10,31]

### 4.5. Limitations of the Study and Perspectives

Some limitations of this study should be noted. The use of male C57BL/6J mice, despite evidence of sex-specific responses to HFD-induced neuroinflammation [1,32], is a constraint, as females exhibit greater resilience through estrogen-mediated mechanisms [1]. Therefore, while our results provide a robust map of L-alliin’s effects in males, future studies including female cohorts are necessary to determine whether these region-specific modulatory patterns—and the striking invariance of hippocampal TNF-α observed here—are conserved across sexes or influenced by hormonal status.

Additionally, mRNA assays were not validated at the protein level, and the single time-point assessment may have missed differential cytokine kinetics, such as earlier TNF-α peaks than IL-6. Furthermore, the absence of direct blood–brain barrier (BBB) integrity measures, such as claudin and occludin quantification, and the lack of functional assays, including behavioral or electrophysiological tests, further constrain interpretations beyond biochemical effects.

These limitations, however, do not detract from the study’s foundational insights but instead highlight opportunities for future research. Including female cohorts could reveal conserved or hormone-influenced modulatory patterns, such as the invariant hippocampal TNF-α response observed here. Investigating dose–response relationships, chronic paradigms, protein validation, and multi-timepoint profiling would refine L-alliin’s therapeutic profile, while incorporating BBB assessments and functional readouts, such as cognitive behavioral tests, would connect cytokine modulation to broader CNS benefits and enhance translational potential.

Overall, these findings position L-alliin as a promising nutraceutical for mitigating obesity-related neuroinflammation and cytokine imbalances, demonstrating its ability to normalize exaggerated responses in metabolically stressed regions, such as the hypothalamus, while selectively reshaping profiles elsewhere. By illustrating regional precision in CNS immunomodulation, this work advances refined strategies for immunometabolic interventions in metabolic syndrome and neurodegeneration, underscoring the value of brain-region-specific approaches in bioactive compound therapeutics.

## 5. Conclusions

These findings position L-alliin as a promising candidate for targeting obesity-related neuroinflammation and cytokine imbalance. Its ability to normalize exaggerated inflammatory responses in metabolically stressed circuits, such as the hypothalamus, while selectively reshaping cytokine profiles in other regions, suggests a potential therapeutic role in early neuroinflammatory states associated with high-fat diets, metabolic syndrome, and increased susceptibility to neurodegeneration.

By demonstrating that nutraceutical compounds can act with regional precision in the CNS, this work paves the way for more refined immunometabolic intervention strategies. It underscores the importance of considering brain-region–specific inflammatory architectures when designing future therapeutic approaches with bioactive compounds.

## Figures and Tables

**Table 1:** Cross-Region Comparison of the Three Brain Regions from two-way ANOVA analysis (* *p* < 0.05, ** *p* < 0.01).

Gene	Factor	Hypothalamus	Frontal Cortex	Hippocampus
IL-1β	Diet	0.434	0.404	0.051
	Treatment	0.032 *	0.053	0.063
	Interaction	0.131	0.562	0.002 **
IL-6	Diet	0.950	0.121	0.917
	Treatment	0.132	0.084	0.339
	Interaction	0.277	0.265	0.045 *
TNF-α	Diet	0.411	0.356	0.612
	Treatment	0.091	0.020 *	0.600
	Interaction	0.090	0.030 *	0.666
CCL2	Diet	0.913	0.257	0.636
	Treatment	0.192	0.258	0.067
	Interaction	0.206	0.275	0.014 *

## Data Availability

The original contributions presented in this study are included in the article and Supplementary Material. Further inquiries can be directed to the corresponding authors.

## Supplementary Materials

The following supporting information can be downloaded at: https://www.mdpi.com/article/10.3390/brainsci16020243/s1.

## Abbreviations

The following abbreviations are used in this manuscript:

ACTB	β-actin
BBB	Blood–brain barrier
CA1	Cornu Ammonis 1 (hippocampal subfield)
CCL2	Chemokine (C-C motif) ligand 2
CNS	Central nervous system
DIO	Diet-induced obesity
DG	Dentate gyrus
ER	Endoplasmic reticulum
FFAR3	Free fatty acid receptor 3
HFD	High-fat diet
ICAM-1	Intercellular adhesion molecule 1
IKKβ	IκB kinase beta
IL-1β	Interleukin-1 beta
IL-6	Interleukin-6
i.p.	Intraperitoneal
LPS	Lipopolysaccharide
mRNA	Messenger ribonucleic acid
NF-κB	Nuclear factor kappa-light-chain-enhancer of activated B cells
qPCR	Quantitative polymerase chain reaction
RT-qPCR	Reverse-transcription quantitative polymerase chain reaction
STD	Standard diet
TLR4	Toll-like receptor 4
TNF-α	Tumor necrosis factor alpha
