# Supplementary material for: L-Alliin Modulates Brain Region-Specific Neuroinflammatory Responses to Lipopolysaccharide in Diet-Induced Obese Mice
Source: Brain Sci. 2026 Feb 22;16(2):243. doi: 10.3390/brainsci16020243 (PMC12938804; doi:10.3390/brainsci16020243)
Supplement: Supplementary file 1 [file brainsci-16-00243-s001.zip › brainsci-4113716-supplementary.pdf]

L-Alliin Modulates Brain Region-Specific Neuroinflammatory Responses to Lipopolysaccharide in Diet-Induced Obese Mice

Celia González-Castillo[1], Daniel Ortuño-Sahagún[2], Carolina Guzmán-Brambila[1], Daniel Torres-Reyes[3], Lucrecia Carrera-Quintanar[3,4,*] and Oscar Arias-Carrión[5,6,*]

File S1

Two-Way ANOVA Results for Gene Expression in Frontal Cortex

The analysis was performed using summary statistics (mean ΔCT ± SD) for each group, n=10 per group and using β-actin as the reference.

The tables below show the ANOVA results for each gene, including sum of squares (SS), degrees of freedom (df), F-statistic, and p-value. Significance notations: *p < 0.05; **p < 0.01; ***p < 0.001; ****p < 0.0001.

**IL1β**				
Source	SS	df	F	p-value
Diet	5.148	1	0.714	0.404
Treatment	28.883	1	4.005	0.053
Diet × Treatment	2.475	1	0.343	0.562
Residual	259.604	36	-	-
**IL-6**				
Source	SS	df	F	p-value
Diet	1.533	1	2.519	0.121
Treatment	1.923	1	3.161	0.084
Diet × Treatment	0.781	1	1.284	0.265
Residual	21.901	36	-	-
**TNF-α**				
Source	SS	df	F	p-value
Diet	1.236	1	0.873	0.356
Treatment	8.363	1	5.909	0.020 *
Diet × Treatment	7.217	1	5.099	0.030 *
Residual	50.948	36	-	-
**CCL2**				
Source	SS	df	F	p-value
Diet	3.516	1	1.327	0.257
Treatment	3.505	1	1.323	0.258
Diet × Treatment	3.260	1	1.231	0.275
Residual	95.373	36	-	-

Two-Way ANOVA Results for Gene Expression in Hippocampus

**IL-1β**				
Source	SS	df	F	p-value
Diet	8.100	1	4.082	0.051
Treatment	7.293	1	3.676	0.063
Diet × Treatment	23.256	1	11.721	0.002 **
Residual	71.429	36	-	-
**IL-6**				
Source	SS	df	F	p-value
Diet	0.011	1	0.011	0.917
Treatment	0.918	1	0.938	0.339
Diet × Treatment	4.212	1	4.302	0.045 *
Residual	35.250	36	-	-
**TNF-α**				
Source	SS	df	F	p-value
Diet	4.051	1	0.263	0.612
Treatment	4.310	1	0.279	0.600
Diet × Treatment	2.932	1	0.190	0.666
Residual	555.515	36	-	-
**CCL2**				
Source	SS	df	F	p-value
Diet	0.903	1	0.228	0.636
Treatment	14.102	1	3.568	0.067
Diet × Treatment	26.358	1	6.669	0.014 *
Residual	142.271	36	-	-

Two-Way ANOVA Results for Gene Expression in hypothalamus

IL-1β				
Source	SS	df	F	p-value
Diet	3.832	1	0.627	0.434
Treatment	30.380	1	4.974	0.032 *
Diet × Treatment	14.568	1	2.385	0.131
Residual	219.872	36	-	-
IL-6				
Source	SS	df	F	p-value
Diet	0.007	1	0.004	0.950
Treatment	4.277	1	2.380	0.132
Diet × Treatment	2.190	1	1.219	0.277
Residual	64.692	36	-	-
TNF-α				
Source	SS	df	F	p-value
Diet	2.460	1	0.693	0.411

Treatment	10.712	1	3.017	0.091
Diet × Treatment	10.754	1	3.028	0.090
Residual	127.841	36	-	-
CCL2				
Source	SS	df	F	p-value
Diet	0.072	1	0.012	0.913
Treatment	10.609	1	1.771	0.192
Diet × Treatment	9.940	1	1.659	0.206
Residual	215.638	36	-	-
